# Combined TCBS and CHROMagar Analyses Allow for Basic Identification of *Vibrio vulnificus* within a 48 h Incubation Period in the Coastal Baltic Sea

**DOI:** 10.3390/microorganisms12030614

**Published:** 2024-03-19

**Authors:** Conor Christopher Glackin, Susann Dupke, Thota Sharath Chandra, David Riedinger, Matthias Labrenz

**Affiliations:** 1 Leibniz Institute for Baltic Sea Research Warnemünde (IOW), Seestraße 15, 18119 Rostock, Germany; conor.glackin@io-warnemuende.de (C.C.G.); sharathota25@gmail.com (T.S.C.); david.riedinger@io-warnemuende.de (D.R.); 2 Robert Koch-Institute (RKI), Centre for Biological Threats and Special Pathogens, Highly Pathogenic Microorganisms, Seestraße 10, 13353 Berlin, Germany; dupkes@rki.de; 3 Marine Research Institute, Klaipeda University, H. Manto 84, LT-92294 Klaipeda, Lithuania

**Keywords:** TCBS agar, CHROMagar™ Vibrio, *Vibrio* spp. identification, *Vibrio vulnificus*, Baltic Sea coastline

## Abstract

With rising infection rates in recent years, *Vibrio vulnificus* poses an increasing threat to public safety in the coastal brackish Baltic Sea. It is therefore important to monitor this organism and assess the *V. vulnificus* infection risk on a more regular basis. However, as the coastline of the Baltic Sea is 8000 km long and shared by nine nations, a convenient, fast, inexpensive, yet efficient *V. vulnificus* identification method is essential. We evaluated the effectiveness of a two-step agar-based approach consisting of successive *Vibrio* isolation and cultivation on thiosulphate-citrate-bile salt sucrose (TCBS) agar and CHROMagar™ *Vibrio* for *V. vulnificus* in comparison with *V. cholerae*, *V. parahaemolyticus*, and *V. alginolyticus*. Our study contains isolates from water and sediment across a broad expanse of the Baltic Sea including 13 locations and two different summers, the time of year during which *Vibrio* infections are usually much more frequent. Confirmation of isolate species identity was carried out using molecular analyses. The two-step agar plating method performed well across different locations and timeframes in correctly identifying *V. vulnificus* by more than 80%, but the sensitivity in other *Vibrio* species varied. Thus, our approach yielded promising results as a potential tool for early *V. vulnificus* detection across a broad timeframe and transect of the Baltic Sea and potentially other brackish environments.

## 1. Introduction

Bacteria of the genus *Vibrio* are ubiquitous members of marine ecosystems and occur in coastal, estuarine, brackish, and freshwaters as well as in sediments, often in association with higher organisms [1]. The genus consists of more than 130 species and around a dozen of these have been demonstrated to be human pathogens [2,3,4,5,6]. From these, *Vibrio cholerae* serotypes O1/O139 can cause the well-known disease cholera. Beside those two, the most important potentially pathogenic non-cholera causing *Vibrio* species are the ‘big four’ species, consisting of *V. cholerae* [different non-O1/O139 serotypes], *V. vulnificus*, *V. parahaemolyticus*, and *V. alginolyticus* [7]. These organisms are common pathogens present in marine and estuarine waters, sediment, or plankton and can cause infections in humans which are usually associated with the consumption of raw or undercooked shellfish or by wound infections in marine or brackish water. Their characteristic disease patterns are gastroenteritis (*V. cholerae*, *V. vulnificus*, and *V. parahaemolyticus*), ear infections (*V. cholerae* and *V. alginolyticus*), wound infections (*V. vulnificus*, *V. cholerae*, *V. alginolyticus*, and *V. parahaemolyticus*), or sepsis (*V. vulnificus*) [2]. Increasing water temperatures can lead to both the increased abundance of *Vibrio* spp. and the occurrence of accordant potential pathogenic *Vibrio* species. Importantly, and this differentiates *Vibrio* spp. in general from the major foodborne pathogens *Salmonella*, *Listeria*, *Escherichia coli* O157, and *Campylobacter*, it has been shown for the USA between 2006 and 2013 that it is the only group currently increasing in occurrence [8]; thus, the behaviour or appearance of members especially of *Vibrio* spp. change in the current environmental conditions. Consequently, with the rise in global sea surface temperatures, there is growing concern about the potential impact of these changes on *Vibrio* populations and the associated public health risks [9].

*V. vulnificus* infections of humans are often severe [>90% of all cases] and have mortality rates as high as 50%, especially in immunodeficient individuals [2,7,10]. *V. vulnificus* blooms have been recorded on numerous occasions [11,12,13] and it is known that its optimal growth conditions involve water temperatures exceeding around 18 °C and salinity levels ranging from 5 to 25 practical salinity units (PSU) [14]. This provides *V. vulnificus* with optimal growth conditions in the Baltic Sea. The Baltic Sea is a semi-enclosed marginal sea of the Atlantic located in northern Europe, with a coastline of approximately 8000 km and covering an area of 415,266 km^2^. Saline inflows through the North Sea produce a 2000 km long lateral surface salinity gradient throughout the whole Baltic Sea, ranging from high salinities [>25] in the transition zone of the Kattegat to low salinities [<5] in the Gulf of Bothnia [15]. The Baltic Sea faces one of the highest warming rates in marine ecosystems worldwide; thus, it is considered a high-risk environment for *Vibrio* infections [16]. Indeed, infection numbers have increased significantly along the Baltic coast in recent years, particularly during heatwaves [13]. During the extremely warm summers of 1994, 2003, and 2006, for instance, a plethora of reports emerged documenting *Vibrio*-associated wound infections linked to recreational exposure in this area and included numerous fatalities [17]. Besides tourists and travellers, the Baltic Sea has almost 30 million people living within 50 km of its coastline with an increasing susceptible risk [18]. This highlights the need to delve into the development of *Vibrio* monitoring tools or early warning systems for *Vibrio* occurrences in the Baltic Sea.

Crucial for the understanding of *Vibrio* spp. distribution is an accurate identification of *Vibrio* species that most frequently cause infections in humans, but especially of the most harmful *V. vulnificus*. Pinpointing a timeframe of increased risk of *Vibrio* infection is the next step in curbing the increasing numbers of infections and also has wide-ranging ecological and economic effects [2,19]. In the context of the Baltic Sea, where diverse *Vibrio* species coexist, precise as well as simple and cost-effective identification becomes more critical. Established methods, such as isolation on agar plates, already exist and have been widely used for *Vibrio* identification [20,21,22]. However, often these methods may lack the specificity and sensitivity required to distinguish between closely related species [21] or require higher media and labour costs [23]. TCBS was one of the first selective media used for the isolation of *Vibrios* [24] and is widely used to isolate *Vibrio* from environmental samples, including the Baltic Sea [25,26]. In contrast to other *Vibrio* selective media, cellobiose-polymyxin B-colistin agar and its modified formulas, modified cellobiose-polymyxin B-colistin agar and cellobiose-colistin agar, TCBS is commercially available and is less time-consuming, requiring only a boiling step [20].

Thus, the objective of this study was to test a two-plate thiosulphate-citrate-bile salt sucrose [TCBS] and CHROMagar™ cultivation approach as an easy, cheap, and efficient species-specific tool to identify potentially pathogenic *Vibrio* in the Baltic Sea. Specification was performed on *V. cholerae*, *V. parahaemolyticus*, *V. vulnificus*, and *V. alginolyticus* identifications, with a broader spatial scale for *V. vulnificus*. Cultivation-based analyses were validated by *Vibrio* spp. specific multiplex-PCR or genus-identifying sequencing on various spatiotemporal levels of the Baltic Sea.

## 2. Materials and Methods

### 2.1. Sampling Areas

**Figure 1:**
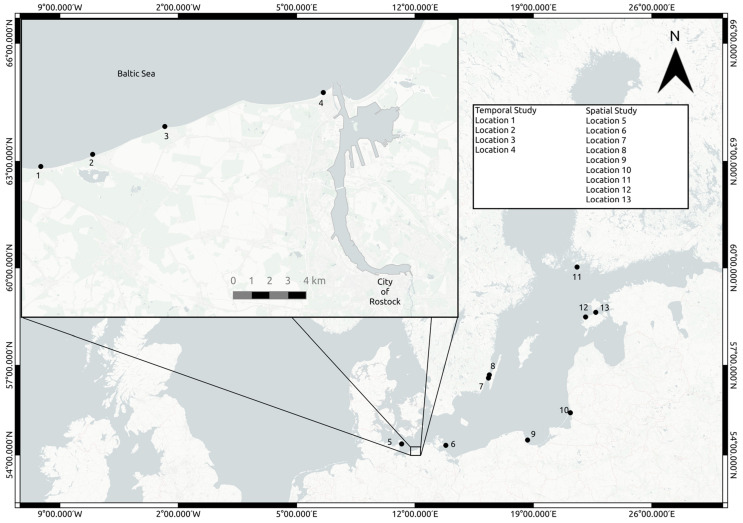
Map of the study area in the Baltic Sea. Temporal sampling stations were 1–4 and spatial sampling stations were 5–13.

To evaluate the presence of potentially pathogenic *Vibrio* in the Baltic Sea based on the combined agar identification approach, two Baltic Sea monitoring campaigns took place, one in 2021 and one in 2022, incorporating a total of 13 sampling stations. A temporal study took place at four locations (Figure 1; locations 1–4, Latitude and Longitude: [54.14636° N, 11.84315° E], [54.15148° N, 11.88636° E], [54.16666° N, 11.96379° E], [54.18248° N, 12.07630° E]) on a 17 km stretch of the northern German coastline across eleven weeks (4 July 2022 to 15 September 2022) and documented *V. vulnificus*, *V. parahaemolyticus*, *V. cholera*, and *V. alginolyticus.* A spatial study focused on *V. vulnificus* abundance over an almost 1000 km range in the Baltic Sea. It took place from 26 July 2021 to 1 September 2021 and encompassed nine sites along the coastlines of Germany, Poland, Finland, and Estonia (Figure 1; locations 5–13, see Supplementary File S3 for coordinates). 

### 2.2. Environmental Sampling

**Figure 2:**
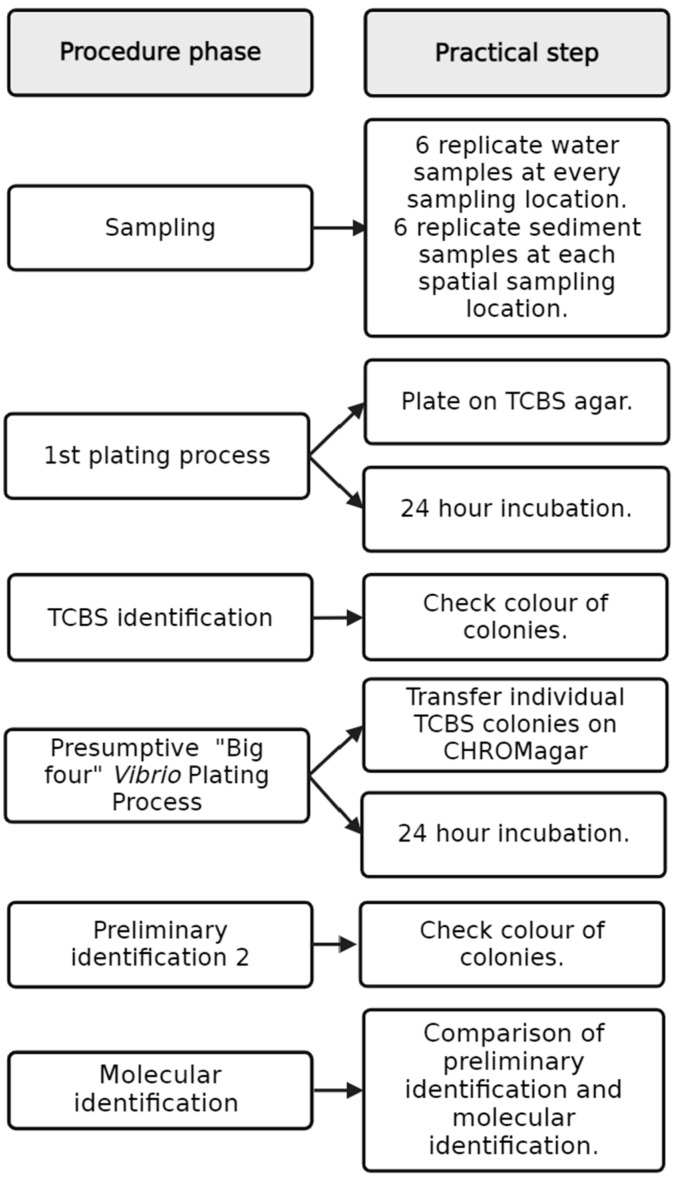
Workflow of the spatiotemporal studies.

Water samples were collected in both campaigns, whereas sediment samples were exclusively gathered during the spatial approach. The workflow from the sampling location to molecular identification and comparison is documented in Figure 2. In the temporal study, surface water (less than 50 cm from surface) was collected at a water depth of around 1 m in 6 replicates. At each station, 15 mL Falcon tubes were dipped into the water with rubber gloves. In Heiligendamm (location 1), the samples were taken 150 m from the shore and ca. 3 m depth and were taken every Tuesday at the same time for the duration of the study. In Börgerende (location 2), Nienhagen (location 3), and Warnemünde (location 4), the samples were taken on the beach at around 1 m depth each Monday and Thursday, for harmonisation always three hours after sunrise, for the duration of the study. Further information on these sampling sites can be found in Supplementary File S2. The spatial study sampling sites consisted of coastal locations along the Baltic Sea (Riedinger et al. in revision. For the spatial study, six replicate water samples were collected by SCUBA divers ca. 20 cm above the sediment with 100 mL syringes and six replicates of the top 1 cm of sediment were collected with 50 mL Falcon tubes. Environmental data of the spatial station are available in Supplementary File S3 and at IOWMeta (doi.io-warnemuende.de/10.12754/data-2023-0010).

All samples were transferred to a 4 °C cooler immediately and stored (maximally 8 h) until processing. Associated physical parameters salinity, temperature, and water depth were measured using a CTD48M (Sea & Sun Technology, Trappenkamp, Germany) during sampling at all stations. 

### 2.3. Vibrio spp. Isolation and Culture-Based Identification

For *Vibrio* spp. isolation and identification, thiosulfate citrate bile sucrose (TCBS) agar (Merck KGaA, Darmstadt, Germany) as well as chromogenic agar selective for *Vibrio* spp. (CHROMagar™, Paris, France) were used. Agar plates were prepared according to the manufacturer’s protocols. For the isolation of the *Vibrio* spp. from the sediment, the overlying water in the Falcon tube was removed, and after homogenisation, a subsample of 10 g (dry-weight determined accurately after lyophilization) was taken from each sample, and in new sterile 50 mL Falcon tubes, 40 mL of double sterile filtered station water was added. Through five ultrasonic pulses of 10 s at 25% capacity at 5 s intervals from the Bandelin SONOPULS HD 2200.2 (Bandelin, Berlin, Germany), sediment-attached bacteria were detached. Following vortexing and sediment settling, water aliquots of 50, 100, or 200 μL were spread on TCBS agar in six biological replicates. For direct plating, each water sample was thoroughly shaken, and 200 μL of the sample was aseptically inoculated onto a TCBS agar plate, which was subsequently evenly spread across the surface. For indirect plating, 2 mL of each water sample was filtered through a 0.22 polycarbonate filter (Isopore™, Merck Millpore Ltd., Cork, Ireland) and this filter was placed on the agar plates. After incubation for 24 h at 37 °C, colonies were quantified and preliminarily categorised according to Table 1.

The preliminarily identified *Vibrio* colonies on TCBS were restreaked onto a ¼ wedge of CHROMagar™ Vibrio plates and incubated for 24 h at 37 °C. The final culture-based identification was documented according to the combined colours of colonies grown on TCBS agar and CHROMagar™ Vibrio (Table 1).

The colonies of presumptive *V. cholerae*, *V. parahaemolyticus*, *V. vulnificus*, and *V. alginolyticus* were suspended in 1 mL of Marine Broth (Roth, Germany). The mixture was left for 24 h at 37 °C. Subsequently, 200 μL of this culture was combined with 300 μL of 50% glycerol, yielding a final concentration of 30% glycerol. These isolates were shock frozen in liquid nitrogen and stored at −80 °C. For recovery, strains were re-cultured either on Columbia agar at 37 °C or in the case of no growth on Difco™ Marine Agar (BD Diagnostics, Sparks, MD, USA) at 28 °C for 24–48 h. 

### 2.4. Molecular Identification of Vibrio spp.

Genomic DNA from *Vibrio* spp. colonies was extracted using the DNeasy Blood and Tissue Kit (Hilden, Germany) according to the following protocol.

A previously frozen bacterial isolate was recultivated on Columbia agar or Difco™ Marine Agar and incubated for 24–48 h. For DNA extraction, an inoculation loop full of colony material was removed by tapping five to ten single colonies. The collected material was transferred into 180 µL ATL buffer and mixed homogeneously by pipetting up and down. Then, 20 µL proteinase K was added, followed by incubation of the sample for 60 min at 56 °C and shaking at 450 rpm in a thermomixer. Subsequently, 200 µL AL buffer was added, and the sample was mixed again by pipetting up and down followed by incubation for another 10 min at 70 °C and shaking at 450 rpm. Further processing was carried out according to the manufacturer’s instructions. The purified DNA was eluted in two steps with 100 µL of elution buffer EB by centrifugation at 600 rpm for 1 min each, so that a total of 200 µL DNA eluate was generated. The DNA was stored at 4 °C. 

The primer and probe sequences for the three multiplex real-time PCR systems are shown in Table 2. The localisation of the primers and probes for *V. cholerae* detection correspond to the sequence of the superoxide dismutase (*sodB*) gene of *V. cholerae* NCTC8457 [GenBank AAWD01000215], as well as the sequence of the cholera toxin (*ctxA*) gene of *V. cholerae* strain B [GenBank AY376267]. The localisation of the primers and probes for *V. parahaemolyticus* detection correspond to the sequence of the toxin regulator (*toxR*) gene of *V. parahaemolyticus* strain KP34 [GenBank DQ845170] and for *V. vulnificus* detection to the sequence of the cytolysin–hemolysin (*vvhA*) gene [GenBank AY046900]. Amplicon lengths for each primer were as follows: 145 bp for *sodB*, 116 bp for *ctxA*, 114 bp for *toxR*, and 118 bp for *vvhA*. In addition, isolates were screened for *V. cholerae* serogroups O1 and O139 [27].

Any bacterial isolate which could not be classified based on the PCR targeting specific markers of *V. cholerae*, *V. vulnificus*, or *V. parahaemolyticus* was subjected to RNA polymerase beta subunit (*rpoB*) sequence determination. Species identification using PCR-based amplification of the *rpoB* gene and analysis of the products were performed as described earlier in Tarr et al. [28] and Schirmeister et al. [29].

Finally, 16S rDNA fragment sequencing was performed on the seven bacterial isolates that could not be identified using *rpoB* gene sequencing. The *rpoB* as well as the 16S rRNA gene fragment sequences were aligned with nucleotide sequences in the GenBank database using the Basic Local Alignment Search Tool (BLAST) search algorithm. 

### 2.5. Statistical Analyses

To test for sampling days that could be considered outliers, the Interquartile Range method was implemented in R statistical package version 4.3.2 using ggplot2:: geom_boxplot.

## 3. Results

### 3.1. Environmental Parameters

For the duration of the temporal study, the temperature ranged between 15 °C and 23 °C and salinity ranged between 9 and 17 PSU in locations 1–4. In the spatial study (locations 5–13), the temperature ranged between 19 °C and 21 °C and salinity ranged between 6 and 10 PSU. A summary of environmental parameters from the temporal study and spatial study can be found in Supplementary Files S1 and S3. 

### 3.2. Identification of Bacterial Isolates on TCBS Agar and CHROMagar™

**Figure 3:**
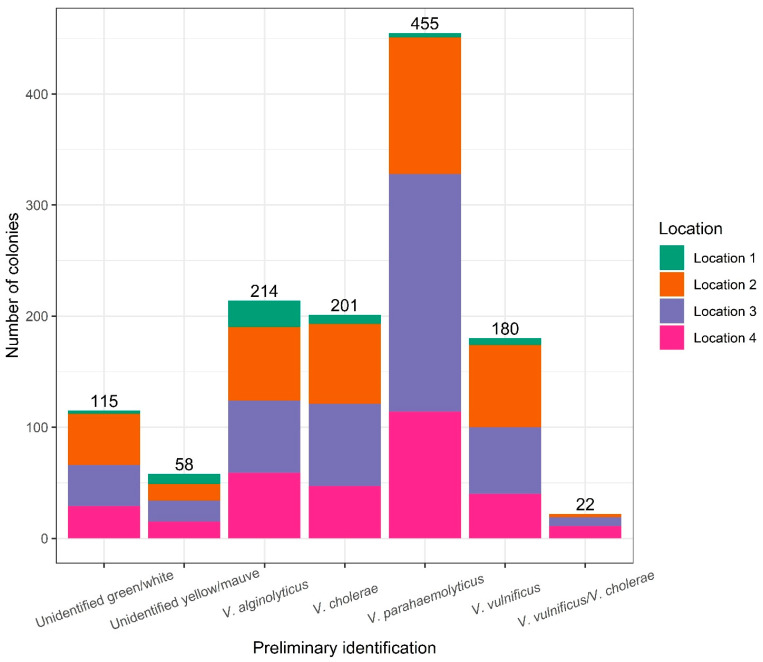
Total number of colonies isolated during the temporal study. The different colours indicate the location. The preliminary identification is based on the combined TCBS/CHROMagar Vibrio colour code (see Table 1).

In the temporal study, a total of 1245 colonies were cultured and isolated on TCBS agar and transferred to CHROMagar™ Vibrio plates. Based on the combined colour code identification, 455 of these colonies (37%) were presumed to be *V. parahaemolyticus* whilst 214 (17%), 201 (16%), and 180 (14%) were presumed to be *V. alginolyticus*, *V. cholerae*, and *V. vulnificus*, respectively (Figure 3). The remaining 195 colonies (16%) were mixed cultures or unidentified using the colour code for species identification on TCBS and CHROMagar Vibrio (Figure 3). The vast majority of colonies originated from water samples taken at locations 2, 3, and 4, with 399, 477, and 315, respectively. The remaining 54 colonies were isolated from location 1 water samples which showed a considerably lower *Vibrio* spp. abundance.

**Figure 4:**
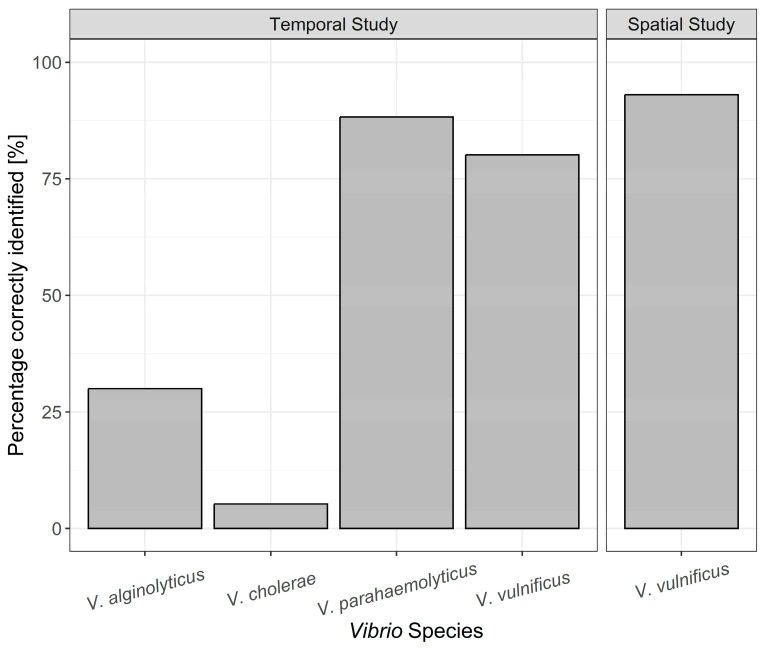
*Vibrio* species correctly assigned using the combined TCBS and CHROMagar Vibrio identification method and confirmed using molecular analysis.

Molecular verification of the culture-dependent *Vibrio* spp. identifications yielded very different values for the individual species. While *V. parahaemolyticus* and *V. vulnificus* were correctly identified with values above 80% by the two-plate TCBS agar/CHROMagar™ Vibrio approach, it was considerably lower for *V. alginolyticus* with 30%. This method also showed poor predictive ability with a value of 5% identification for *V. cholerae* (Figure 4). Of the misidentified presumptive *V. cholerae* colonies, 36.5 % belonged to *V. aestuarianus* and 51.0% to *V. diazotrophicus* based on molecular analyses. *V. vulnificus* colonies were cultured in each of the eleven weeks and were consistently isolated from samples across the three beach locations (locations 2–4).

**Figure 5:**
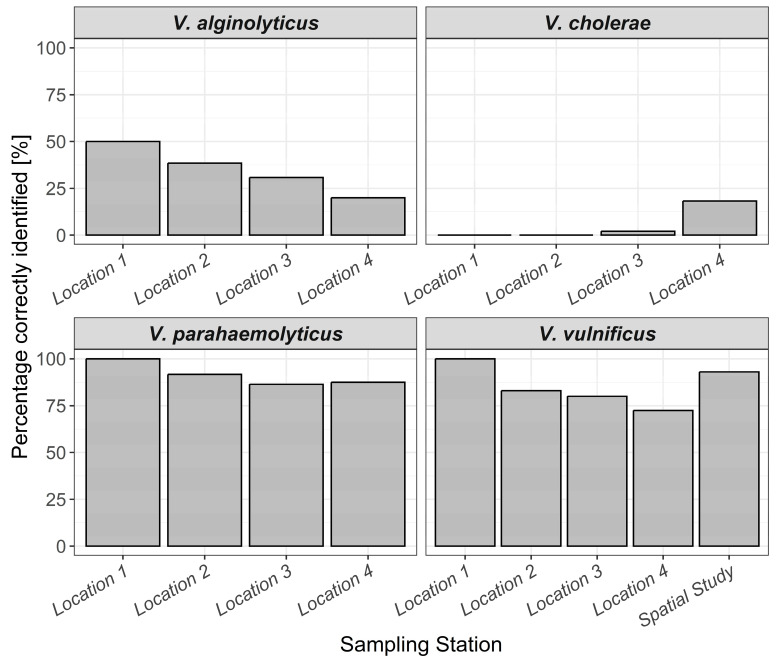
*Vibrio* species correctly assigned using the TCBS and CHROMagar Vibrio identification method and confirmed using molecular analysis. Graph is split by species and location.

In the spatial study from 2021, 86 colonies from eight different locations in the Baltic Sea presumed to be *V. vulnificus* were successfully cultured (Supplementary File S3). From these, 93% were accurately identified by the two-plate TCBS agar/CHROMagar™ Vibrio approach (Figure 4). Comparing correctly identified isolates from both the temporal study and spatial study, it became clear that *V. vulnificus* could be regularly identified correctly at a high level using the two-plate method. For *V. parahaemolyticus*, this method also showed high predictive power, correctly identifying 88% of colonies (Figure 4). In contrast, the identification level of *V. alginolyticus* and *V. cholerae* appeared to vary at an already low level (Figure 5). The number of isolates genetically identified in each location is shown in Table S1. 

### 3.3. Highest Vibrio spp. Abundance per Day

**Figure 6:**
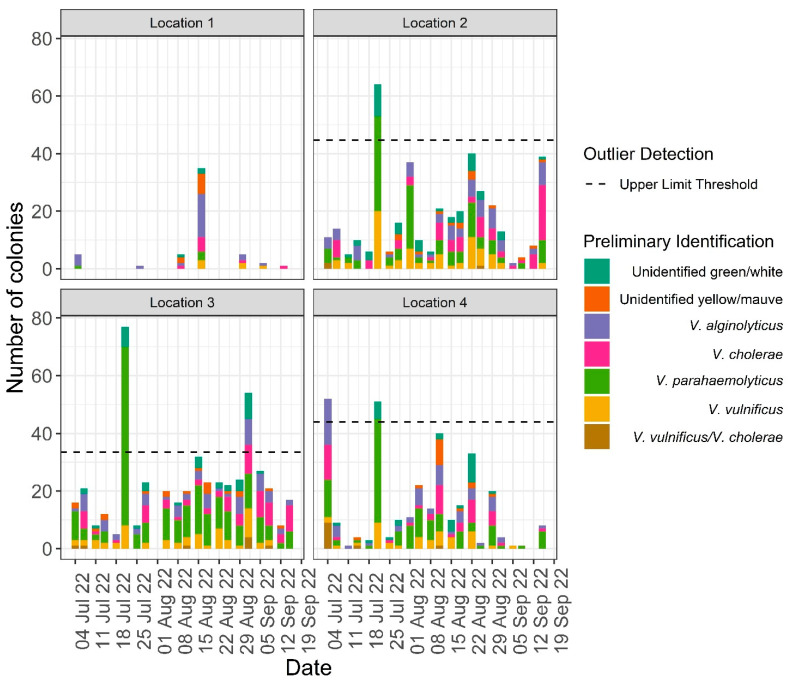
Total species split by location and coloured by presumed identity on TCBS and CHROM-agar Vibrio using the colour coding scheme (see Table 1). Unidentified green/white and unidentified yellow/mauve indicate the colours observed on TCBS and CHROMagar, respectively. *V. vulnificus*/*V. cholerae* refers to colonies that could not definitively be identified as either species using the colour code. Outlier detection using upper boxplot range is shown (Figure S1).

Of the total *Vibrio* spp. colonies isolated, it was found that 96% were extracted from water samples taken from locations 2, 3, and 4. Further investigation highlighted that one day in particular yielded a significantly higher number of colonies at these locations (Figure 6). In total, there were 22 days in which samples were taken at each of these locations and 21 July 2022 accounted for 15% (192 colonies) of the total colonies cultured. This is a sharp rise from the total of 14 colonies cultured across all locations on the 18 July. Of the 37 presumptive *V. vulnificus* samples cultured on this day, 24 were identified using molecular sequencing. A total of 18 of these 24 isolates (75%) were confirmed to be *V. vulnificus*, demonstrating similar predictive results to the overall spatiotemporal analysis within this day.

## 4. Discussion

Routine monitoring for *Vibrio vulnificus* in the Baltic Sea is critical to provide a warning system for the public when the risk of infection is potentially high. This study examined using TCBS agar and CHROMagar™ Vibrio-based *V. vulnificus* identifications to achieve this, spanning across two separate spatiotemporal sampling projects. For evaluation, this agar-based method was also tested on *V. cholerae*, *V. parahaemolyticus*, and *V. alginolyticus* in the temporal study. The two-plate agar method proved to be accurate in identifying *V. vulnificus* across a broad range of locations and timeframes within the Baltic Sea, although it had contrasting results when used on other species of *Vibrio*.

*V. vulnificus* presents the largest threat for open wound infections in the Baltic Sea [30]. In total, 222 *V. vulnificus* samples were genetically identified across twelve locations and two different timeframes in 2021 and 2022, giving a comprehensive overview of this method of identification in the Baltic Sea. The overall correct identification of *V. vulnificus* was 85% and was consistent in correct identification across sampling sites (Figure 5). Thus, our study indicates that the use of TCBS agar followed by CHROMagar™ Vibrio to preliminarily identify *V. vulnificus* in the Baltic Sea produces applicable results.

In recent years, there have been numerous attempts to provide the accurate identification of *V*. *vulnificus* using a culture-based approach, which differ greatly in their success rate. TCBS agar alone has been shown to be not sufficiently selective enough for environmental *Vibrio* samples [31]. Thus, the vast majority of more recent research involves using TCBS agar, CHROMagar™ Vibrio, or Cellobiose polymyxin B colistin (CPC) agar with varying results in environmental samples. The highest performing agar approach for *V. vulnificus* so far was a triple plating method [23], producing a 92.8% accuracy on environmental water and oyster samples. Other comprehensive studies include a study by Froelich et al. [32] where CPC+ was used to culture presumptive *V. vulnificus* on agar. Results showed that the average yearly rate of samples confirmed to be *V. vulnificus* ranged from 0% to 45.7%. In another study by Froelich et al. [33] on oyster meat, they compared four different medium methods for presumptive *V. vulnificus*, resulting in correct identification rates between 44% and 81%, as confirmed by PCR. A study in the Mediterranean using TCBS and CPC found, with 3.7% and 7.6%, much lower accuracy levels of these two agar methods, respectively [34]. 

*V. vulnificus* aside, *V. cholerae*, *V. parahaemolyticus*, and *V. alginolyticus* are responsible for the majority of other *Vibrio* wound infections in the Baltic Sea [11,13]. In stark contrast to *V. vulnificus*, the percentage of *V. cholerae* correctly identified was 5%. This highlights a huge difference in identifying the two species responsible for the most *Vibrio* infections in the Baltic Sea using the two-plate TCBS agar/CHROMagar™ Vibrio approach method. The vast majority of the misidentified bacterial isolates were *V. aestuarianus* and *V. diazotrophicus*, reflecting that the *V. cholerae* colony colours using this agar method are close between the two species. In the case of *V. parahaemolyticus*, the TCBS agar/CHROMagar™ Vibrio yielded high correct identification results, whereas the presumptive *V. alginolyticus* colonies were misidentified the majority of the time, further highlighting the mixed results of this agar identification method on different *Vibrio* species.

### Spatiotemporal Analysis

In the temporal study, 1245 different presumptive *Vibrio* spp. colonies were grown. In terms of overall *Vibrio* spp. cultivated, station 1 had considerably fewer colonies than the other three locations. The notable difference here was the water depth at the sampling areas. Although all samples were surface water, the difference in depth was around 2 m (3 m vs. 1 m) and the distance from the shoreline was 150 m for location 1 and around 10 m for locations 2–4. There are several hypotheses as to why this discrepancy occurred. Numerous environmental parameters, most notably temperature and salinity, have been associated with contributing to *Vibrio* spp. abundance, depending on species, habitat, and geographic location [35,36,37,38,39,40,41,42,43,44]. The similarity in both temperature and salinity in our project suggests that other environmental parameters or processes may have contributed to the change in abundance. Dissolved oxygen [45,46,47], chlorophyll [39,48,49,50], and plankton [39,51,52,53,54] have also been found to be important in the ecology of *Vibrio* spp. Given that location 1 was further from the shore and sediment, it is also possible that turbidity, increased nutrient loads, and increased sediment bacteria resuspension also played a role in the difference in *Vibrio* spp. abundance between location 1 and locations 2–4.

In the three beach locations, the temporal dynamics were similar in both overall colonies cultured and species correctly identified (Figure 4), with the 22 July showing the highest number of colonies cultivated across all locations. This day was an outlier in all three locations with regard to overall colonies cultured, highlighting the consistency in the results along the 17 km stretch of coast where the *Vibrio* spp. summer surveillance took place. Finding a significantly higher number of presumptive *Vibrio* isolates in all locations demonstrates the need to further pinpoint *Vibrio* blooms, given that they are present in the marine environment throughout the summer months. This once again points to the need for a simple and fast *Vibrio* spp. identification option. The establishment of a principal monitoring or even an early warning system for *V. vulnificus* in the Baltic Sea is of paramount importance due to the potential public health risks and ecological consequences associated with this pathogenic bacterium. *V. vulnificus* is a well-recognised human pathogen and infections have been documented in increasing numbers along the Baltic coast in recent years [30]. Tourism and economic impacts associated with the danger of infections have been described recently [5,55]. Current research suggests a correlation between sea temperature and *V. vulnificus* abundance and this has major implications for the Baltic Sea given that it is one of the fastest warming seas in the world [9].

This study introduces valuable insights into the suitability of early detection methods for different *Vibrio* species in the region. The reduction of false positive identification in culture-based methods enhances predictive power but can also significantly decrease the cost of laboratory equipment and procedures that are necessary for molecular identification of *Vibrio* spp. This is especially useful in areas or situations where molecular analysis is not possible or is too time consuming, and the price of cultivating colonies in this study using the two-plate TCBS agar and CHROMagar™ Vibrio approach was less than USD 3 per sample. An unexpected outcome of the agar identification was the high percentage of *V. aestuarianus* and *V. diazotrophicus* misidentified as *V. cholerae* and this suggests that presumptive identification varies between species and that alternative agar methods may be better in identifying these organisms. The development of improved monitoring or an early warning system for *Vibrio* spp. in the Baltic Sea may be produced using an amalgamation of agar methods and other environmental, chemical, and biological parameters to create predictive models.

## 5. Conclusions

Our study documents a quick and straightforward method of isolating presumptive *V. vulnificus* strains using a two-plate TCBS agar and CHROMagar™ Vibrio approach. Similar correct identification results, confirmed by molecular analyses, across various locations and timeframes in two different years suggests that this method can be used as a general marker for further research into this topic in the Baltic Sea and probably other brackish environments. Varying results documented with other *Vibrio* species indicate that this method is not a ‘one size fits all’ approach to *Vibrio* spp. identified and other agar methods may yield more consistent results. 

## Figures and Tables

**Table 1:** Species identification of colony forming units (CFUs) according to colour on TCBS and CHROMagar Vibrio.

TCBS CFU Colour	CHROM CFU Colour	*Vibrio* Preliminary Identification
Green	Blue	*V. vulnificus*
Green	Mauve	*V. parahaemolyticus*
Yellow	Blue	*V. cholerae*
Yellow	White	*V. alginolyticus*/Other *Vibrio* spp.

**Table 2:** List of primers used per species. All primers were originally used in Messelhäusser et al. [27].

Target Species	Primer/Probe	Sequenz (5′–3′)	Localisation
*V. cholerae*	sodB f	AAGACCTCAACTGGCGGTA	276–294
	sodB r	CAGCAAAAGAACCGAATGCT	420–401
	sodB TM	Cy5-GCAGGTTTGGAACCACACTT-BHQ-2	311–330
	ctx f	AGTTCATTTTGGGGTGCTTG	369–388
	ctx r	GGAAACCTGCCAATCCATAA	484–465
	ctx TM	FAM-CATCGTAATAGGGGCTACAGAGA-BHQ-1	400–422
*V. parahaemolyticus*	toxR f	CCAGAAGCGCCAGTAGTACC	149–168
	toxR r	AAACAGCAGTACGCAAATCG	262–243
	toxR TM	FAM-TGTGGCTTCTGCTGTGAATC-BHQ-1	181–200
*V. vulnificus*	vvhA f	ACCAAGTTTGGGGCCTAGAT	389–408
	vvhA r	GCTAAGTTCGCACCACACTG	506–487
	vvhA TM	Cy5-CCGATCGTTGTTTGACCGTA-BHQ-2	440–459

## Data Availability

The original contributions presented in this study are included in the article and the associated Supplementary material. In addition, data associated with the spatial study are available at IOWMeta (doi.io-warnemuende.de/10.12754/data-2023-0010).

## Supplementary Materials

The following supporting information can be downloaded at https://www.mdpi.com/article/10.3390/microorganisms12030614/s1, Information on sampling sites, physical parameters, and CFU identification can be found in Supplementary File S1 and Supplementary File S2. Supplementary File S3 documents the station ID, longitude, latitude, date, sampling depth, temperature, and salinity of the corresponding isolates. Table S1. Colonies per *Vibrio* species from each location of isolates correctly identified on TCBS and CHROMagar and confirmed using molecular analyses. Figure S1. Data are represented as boxplots where the middle line is the median, the lower and upper hinges correspond to the first and third quartiles, the upper whisker extends from the hinge to the largest value no further than 1.5× IQR from the hinge (where IQR is the inter-quartile range), and the lower whisker extends from the hinge to the smallest value at most 1.5× IQR of the hinge. Please see function geom_boxplot in R (ggplot2).

## References

[1] Le Roux F., Blokesch M. (2018). Eco-evolutionary dynamics linked to horizontal gene transfer in Vibrios. Annu. Rev. Microbiol..

[2] Baker-Austin C., Oliver J.D., Alam M., Ali A., Waldor M.K., Qadri F., Martinez-Urtaza J. (2018). *Vibrio* spp. infections. Nat. Rev. Dis. Primers.

[3] Potdukhe T.V., Caffrey J.M., Rothfus M.J., Daniel C.E., Swords M.E., Albrecht B.B., Jeffrey W.H., Waidner L.A. (2021). Viable putative *Vibrio vulnificus* and *parahaemolyticus* in the Pensacola and Perdido Bays: Water column, sediments, and invertebrate biofilms. Front. Mar. Sci..

[4] Ceccarelli D., Amaro C., Romalde J.L., Suffredini E., Vezzulli L. (2019). Vibrio species. Food Microbiology.

[5] Sampaio A., Silva V., Poeta P., Aonofriesei F. (2022). *Vibrio* spp.: Life strategies, ecology, and risks in a changing environment. Diversity.

[6] Thompson F.L., Austin B., Swings J. (2006). The Biology of Vibrios.

[7] Baker-Austin C., Trinanes J., Gonzalez-Escalona N., Martinez-Urtaza J. (2017). Non-cholera Vibrios: The microbial barometer of climate change. Trends Microbiol..

[8] Crim S.M., Iwamoto M., Huang J.Y., Griffin P.M., Gilliss D., Cronquist A.B., Cartter M., Tobin-D’Angelo M., Blythe D., Smith K. (2014). Incidence and trends of infection with pathogens transmitted commonly through food--Foodborne Diseases Active Surveillance Network, 10 U.S. sites, 2006–2013. MMWR Morb. Mortal. Wkly. Rep..

[9] Baker-Austin C., Trinanes J.A., Taylor N.G.H., Hartnell R., Siitonen A., Martinez-Urtaza J. (2013). Emerging *Vibrio* risk at high latitudes in response to ocean warming. Nat. Clim. Chang..

[10] Jones M.K., Oliver J.D. (2009). *Vibrio vulnificus*: Disease and pathogenesis. Infect. Immun..

[11] Amato E., Riess M., Thomas-Lopez D., Linkevicius M., Pitkänen T., Wołkowicz T., Rjabinina J., Jernberg C., Hjertqvist M., MacDonald E. (2022). Epidemiological and microbiological investigation of a large increase in vibriosis, northern Europe, 2018. Euro Surveill..

[12] Baker-Austin C., Trinanes J.A., Salmenlinna S., Löfdahl M., Siitonen A., Taylor N.G.H., Martinez-Urtaza J. (2016). Heat wave-associated Vibriosis, Sweden and Finland, 2014. Emerg. Infect. Dis..

[13] Brehm T.T., Berneking L., Sena Martins M., Dupke S., Jacob D., Drechsel O., Bohnert J., Becker K., Kramer A., Christner M. (2021). Heatwave-associated *Vibrio* infections in Germany, 2018 and 2019. Euro Surveill..

[14] Oliver J.D. (2015). The biology of *Vibrio vulnificus*. Microbiol. Spectr..

[15] Maar M., Møller E.F., Larsen J., Madsen K.S., Wan Z., She J., Jonasson L., Neumann T. (2011). Ecosystem modelling across a salinity gradient from the North Sea to the Baltic Sea. Ecol. Model..

[16] Belkin I.M. (2009). Rapid warming of large marine ecosystems. Prog. Oceanogr..

[17] Frank C., Littman M., Alpers K., Hallauer J. (2006). *Vibrio vulnificus* wound infections after contact with the Baltic Sea, Germany. Euro Surveill..

[18] New World Encyclopedia Baltic Sea—New World Encyclopedia, 2023UTC. https://www.newworldencyclopedia.org/p/index.php?title=Baltic_Sea&oldid=1122453.

[19] Schütt E.M., Hundsdörfer M.A.J., von Hoyningen-Huene A.J.E., Lange X., Koschmider A., Oppelt N. (2023). First steps towards a near real-time modelling system of *Vibrio vulnificus* in the Baltic Sea. Int. J. Environ. Res. Public Health.

[20] Choopun N., Louis V., Huq A., Colwell R.R. (2002). Simple procedure for rapid identification of *Vibrio cholerae* from the aquatic environment. Appl. Environ. Microbiol..

[21] Di Pinto A., Terio V., Novello L., Tantillo G. (2011). Comparison between thiosulphate-citrate-bile salt sucrose (TCBS) agar and CHROMagar *Vibrio* for isolating *Vibrio parahaemolyticus*. Food Control.

[22] Tamura K., Shimada S., Prescott L.M. (1971). *Vibrio* agar: A new plating medium for isolation of *Vibrio cholerae*. Jpn. J. Med. Sci. Biol..

[23] Williams T.C., Froelich B., Oliver J.D. (2013). A new culture-based method for the improved identification of *Vibrio vulnificus* from environmental samples, reducing the need for molecular confirmation. J. Microbiol. Methods.

[24] Oliver J.D., Hope C.F.A., Adams M.R. (1989). Chapter 17 Culture media for the isolation and enumeration of pathogenic *Vibrio* species in foods and environmental samples. Rapid Methods in Food Microbiology.

[25] Gyraite G., Katarzyte M., Schernewski G. (2019). First findings of potentially human pathogenic bacteria *Vibrio* in the south-eastern Baltic Sea coastal and transitional bathing waters. Mar. Pollut. Bull..

[26] Gomez-Gil B., Roque A., Thompson F.L., Austin B., Swings J. (2006). Isolation, Enumeration, and Preservation of the Vibrionaceae. The Biology of Vibrios.

[27] Messelhäusser U., Colditz J., Thärigen D., Kleih W., Höller C., Busch U. (2010). Detection and differentiation of *Vibrio* spp. in seafood and fish samples with cultural and molecular methods. Int. J. Food. Microbiol..

[28] Tarr C.L., Patel J.S., Puhr N.D., Sowers E.G., Bopp C.A., Strockbine N.A. (2007). Identification of *Vibrio* isolates by a multiplex PCR assay and rpoB sequence determination. J. Clin. Microbiol..

[29] Schirmeister F., Dieckmann R., Bechlars S., Bier N., Faruque S.M., Strauch E. (2014). Genetic and phenotypic analysis of *Vibrio cholerae* non-O1, non-O139 isolated from German and Austrian patients. Eur. J. Clin. Microbiol. Infect. Dis..

[30] Fleischmann S., Herrig I., Wesp J., Stiedl J., Reifferscheid G., Strauch E., Alter T., Brennholt N. (2022). Prevalence and distribution of potentially human pathogenic *Vibrio* spp. on German North and Baltic Sea coasts. Front. Cell Infect. Microbiol..

[31] Lotz M.J., Tamplin M.L., Rodrick G.E. (1983). Thiosulfate-citrate-bile salts-sucrose agar and its selectivity for clinical and marine vibrio organisms. Ann. Clin. Lab..

[32] Froelich B.A., Weiss M.J., Noble R.T. (2014). The evaluation of four recent culture-based methods for the isolation and enumeration of *Vibrio vulnificus* bacteria from oyster meat. J. Microbiol. Methods.

[33] Froelich B.A., Williams T.C., Noble R.T., Oliver J.D. (2012). Apparent loss of *Vibrio vulnificus* from North Carolina oysters coincides with a drought-induced increase in salinity. Appl. Environ. Microbiol..

[34] Arias C.R., Aznar R., Pujalte M.J., Garay E. (1998). A comparison of strategies for the detection and recovery of *Vibrio vulnificus* from marine samples of the western Mediterranean coast. Syst. Appl. Microbiol..

[35] Colwell R.R. (1996). Global climate and infectious disease: The cholera paradigm. Science.

[36] DePaola A., Nordstrom J.L., Bowers J.C., Wells J.G., Cook D.W. (2003). Seasonal abundance of total and pathogenic *Vibrio parahaemolyticus* in Alabama oysters. Appl. Environ. Microbiol..

[37] Grimes D.J., Johnson C.N., Dillon K.S., Flowers A.R., Noriea N.F., Berutti T. (2009). What genomic sequence information has revealed about *Vibrio* ecology in the ocean—A review. Microb. Ecol..

[38] Huq A., West P.A., Small E.B., Huq M.I., Colwell R.R. (1984). Influence of water temperature, salinity, and pH on survival and growth of toxigenic *Vibrio cholerae* serovar 01 associated with live copepods in laboratory microcosms. Appl. Environ. Microbiol..

[39] Johnson C.N., Flowers A.R., Noriea N.F., Zimmerman A.M., Bowers J.C., DePaola A., Grimes D.J. (2010). Relationships between environmental factors and pathogenic Vibrios in the Northern Gulf of Mexico. Appl. Environ. Microbiol..

[40] Kelly M.T. (1982). Effect of temperature and salinity on *Vibrio* (Beneckea) vulnificus occurrence in a Gulf Coast environment. Appl. Environ. Microbiol..

[41] Stauder M., Vezzulli L., Pezzati E., Repetto B., Pruzzo C. (2010). Temperature affects *Vibrio cholerae* O1 El Tor persistence in the aquatic environment via an enhanced expression of GbpA and MSHA adhesins. Environ. Microbiol. Rep..

[42] Lobitz B., Beck L., Huq A., Wood B., Fuchs G., Faruque A.S., Colwell R. (2000). Climate and infectious disease: Use of remote sensing for detection of *Vibrio cholerae* by indirect measurement. Proc. Natl. Acad. Sci. USA.

[43] Zimmerman A.M., DePaola A., Bowers J.C., Krantz J.A., Nordstrom J.L., Johnson C.N., Grimes D.J. (2007). Variability of total and pathogenic *Vibrio parahaemolyticus* densities in northern Gulf of Mexico water and oysters. Appl. Environ. Microbiol..

[44] Tamplin M., Rodrick G.E., Blake N.J., Cuba T. (1982). Isolation and characterization of *Vibrio vulnificus* from two Florida estuaries. Appl. Environ. Microbiol..

[45] Igbinosa E.O., Obi C.L., Okoh A.I. (2011). Seasonal abundance and distribution of *Vibrio* species in the treated effluent of wastewater treatment facilities in suburban and urban communities of Eastern Cape Province, South Africa. J. Microbiol..

[46] Parveen S., Hettiarachchi K.A., Bowers J.C., Jones J.L., Tamplin M.L., McKay R., Beatty W., Brohawn K., Dasilva L.V., DePaola A. (2008). Seasonal distribution of total and pathogenic *Vibrio parahaemolyticus* in Chesapeake Bay oysters and waters. Int. J. Food Microbiol..

[47] Ramirez G.D., Buck G.W., Smith A.K., Gordon K.V., Mott J.B. (2009). Incidence of *Vibrio vulnificus* in estuarine waters of the south Texas Coastal Bend region. J. Appl. Microbiol..

[48] Caburlotto G., Haley B.J., Lleò M.M., Huq A., Colwell R.R. (2010). Serodiversity and ecological distribution of *Vibrio parahaemolyticus* in the Venetian Lagoon, Northeast Italy. Environ. Microbiol. Rep..

[49] Deter J., Lozach S., Derrien A., Véron A., Chollet J., Hervio-Heath D. (2010). Chlorophyll a might structure a community of potentially pathogenic culturable Vibrionaceae. Insights from a one-year study of water and mussels surveyed on the French Atlantic coast. Environ. Microbiol. Rep..

[50] Julie D., Solen L., Antoine V., Jaufrey C., Annick D., Dominique H.-H. (2010). Ecology of pathogenic and non-pathogenic *Vibrio parahaemolyticus* on the French Atlantic coast. Effects of temperature, salinity, turbidity and chlorophyll a. Environ. Microbiol..

[51] Asplund M.E., Rehnstam-Holm A.-S., Atnur V., Raghunath P., Saravanan V., Härnström K., Collin B., Karunasagar I., Godhe A. (2011). Water column dynamics of *Vibrio* in relation to phytoplankton community composition and environmental conditions in a tropical coastal area. Environ. Microbiol..

[52] Martinez-Urtaza J., Huapaya B., Gavilan R.G., Blanco-Abad V., Ansede-Bermejo J., Cadarso-Suarez C., Figueiras A., Trinanes J. (2008). Emergence of Asiatic *Vibrio* diseases in South America in phase with El Niño. Epidemiology.

[53] Rehnstam-Holm A.S., Godhe A., Härnström K., Raghunath P., Saravanan V., Collin B., Karunasagar I. (2010). Association between phytoplankton and *Vibrio* spp. along the southwest coast of India: A mesocosm experiment. Aquat. Microb. Ecol..

[54] Turner J.W., Good B., Cole D., Lipp E.K. (2009). Plankton composition and environmental factors contribute to *Vibrio* seasonality. ISME J..

[55] Novriadi R. (2016). Vibriosis in aquaculture. Omni Akuatika.

