# Supplementary material for: Combined TCBS and CHROMagar Analyses Allow for Basic Identification of Vibrio vulnificus within a 48 h Incubation Period in the Coastal Baltic Sea
Source: Microorganisms. 2024 Mar 19;12(3):614. doi: 10.3390/microorganisms12030614 (PMC10974389; doi:10.3390/microorganisms12030614)
Supplement: Supplementary file 1 [file microorganisms-12-00614-s001.zip › Supplementary Information.pdf]

Supplementary Information

Temporal study: All figures and graphs contained within the Supplementary Information contain data from the Temporal study. Information on sampling sites, physical parameters, and CFU identification can be found in Supplementary File S1 and Supplementary File S2.
Spatial study: Information on sampling sites, physical parameters, and CFU identification can be found at 10.12754/data-2023-0010. Additionally Supplementary File S3 documents the station ID, longitude, latitude, date, sampling depth, temperature, salinity of the corresponding isolates.

Table S1. Colonies per *Vibrio* Species from each location of isolates correctly identified on TCBS and CHROMagar and confirmed using molecular analyses.

Sampling campaign	*Vibrio* species	Correctly Assigned	Heiligendamm	Börgerende	Nienhagen	Warnemünde	Total
Temporal Study	*V. alginolyticus*	Yes	2	5	4	4	**15**
		No	2	8	9	16	**35**
	V. cholerae	Yes	0	0	1	6	**7**
		No	4	49	46	27	**126**
	V. parahaemolyticus	Yes	1	11	19	14	**45**
		No	0	1	3	2	**6**
	V. vulnificus	Yes	4	44	40	21	**109**
		No	0	9	10	8	**27**
Spatial Study	*V. vulnificus*	Yes	-				**80**
		No					**6**

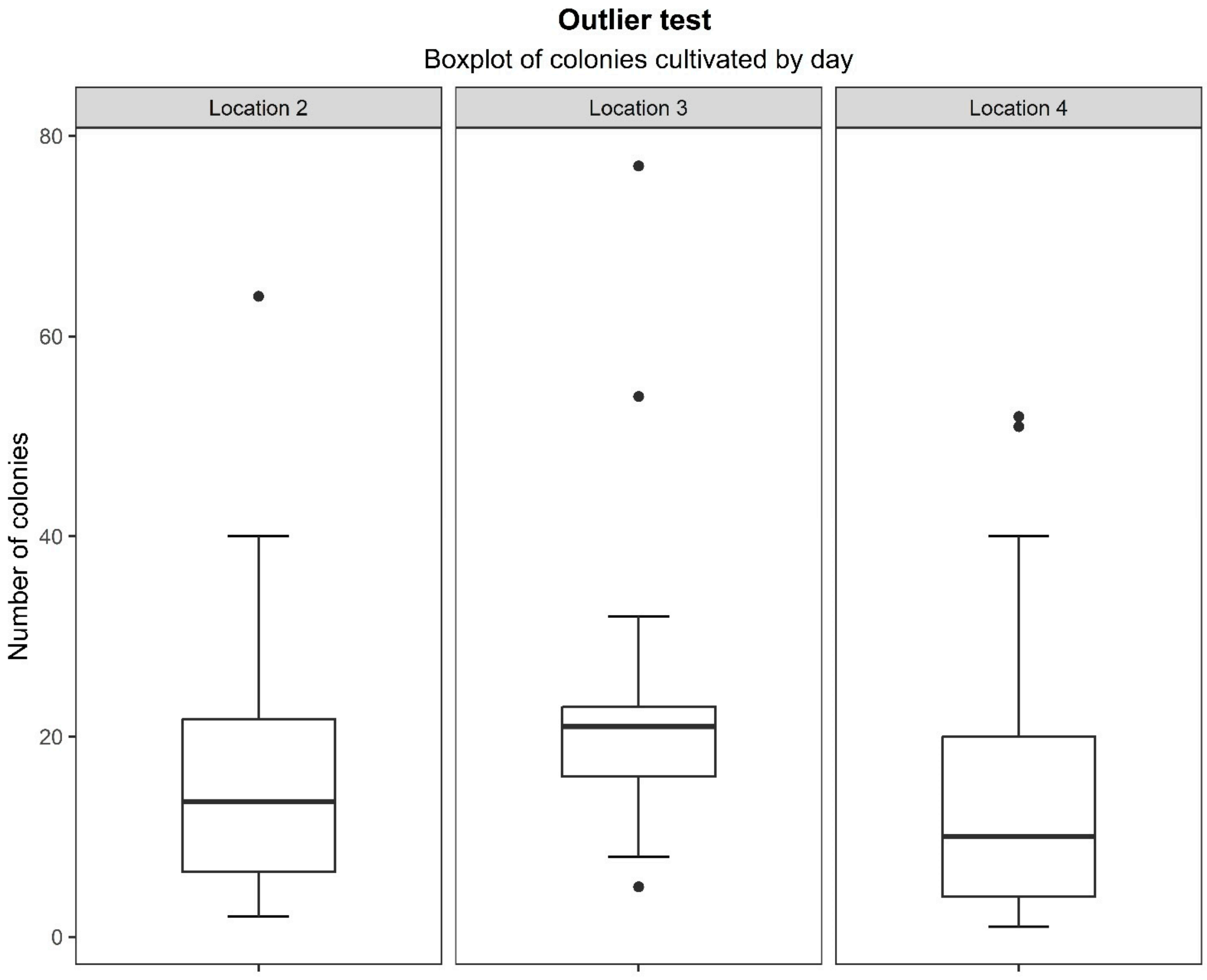

Figure S1. Data are represented as boxplots where the middle line is the median, the lower and upper hinges correspond to the first and third quartiles, the upper whisker extends from the hinge to the largest value no further than 1.5× IQR from the hinge (where IQR is the inter-quartile range) and the lower whisker extends from the hinge to the smallest value at most 1.5× IQR of the hinge. Please see function geom_boxplot in R (ggplot2).
